# A Highly Pathogenic Strain of *Staphylococcus sciuri* Caused Fatal Exudative Epidermitis in Piglets

**DOI:** 10.1371/journal.pone.0000147

**Published:** 2007-01-10

**Authors:** Shixi Chen, Yu Wang, Fuyong Chen, Hanchun Yang, Menghou Gan, Shijun J. Zheng

**Affiliations:** Ministry of Agriculture Key Laboratory of Preventive Veterinary Medicine, College of Veterinary Medicine, China Agricultural University, Beijing, China; University of Liverpool, United Kingdom

## Abstract

*Staphylococcus sciuri* are important human pathogens responsible for endocarditis, peritonitis, septic shock, urinary tract infection, pelvic inflammatory disease and wound infections. However, little information is known regarding the pathogenicity of *S. sciuri* to animals. From the pericardial fluid of a diseased piglet with exudative epidermitis (EE), we isolated a strain of *Staphylococcus* in pure culture. Surprisingly, this isolate was a member of *S. sciuri* rather than *S. hyicus* as identified by its biochemical traits and also by analysis of 23S ribosomal DNA using Internal Transcribed Spacer PCR. In addition, inoculation of newborn piglets with 1×10^10^ CFU of the isolate by oral feeding or intra-muscular injection successfully reproduced EE in piglets, which suggested that the oral intake of the pathogen by the animals is one of the major routes of exposure. These unexpected findings prioritized *S. sciuri* as important zoonotic agents, which may have ramifications for human medicine.

## Introduction

Exudative epidermitis (EE) in pigs, also called Greasy Pig Disease, is a communicable skin disease caused by pathogenic strains of staphylococci. This disease is mostly seen as an acute or peracute infection in suckling and newly weaned piglets although it may occur as a chronic infection in adult [1]–[4]. The diseased piglet develops a generalized exudative epidermitis and may die within a few days due, at least in part, to dehydration. The most commonly seen causative agent for this disease was *Staphylococcus hyicus,* which produced secretory exfoliative toxins as the major factors to cause skin lesions [5]. At least six exfoliative toxins, ExhA through D, ShetA and ShetB, have been described so far [6]–[8] and their existence are species-dependent [9]. In addition to epidermitis, *S. hyicus* may induce mastitis in cow [10], [11], polyarthritis in pig [12], [13] and reproductive failure in sow [14]. Exudative epidermitis shared similar histopathology with the human infection called the *Staphylococcus aureus* scalded skin syndrome (SSSS) in terms of blister formation and exfoliation of the skin caused by splitting of the skin at the granular layer of the epidermis [15], which may be accounted for by the production of similar exfoliative toxins by *S. hyicus* and *S. aureus* [16].

*S. hyicus* is generally recognized as the causative agent of EE, however the other species of Staphylococcus may also cause this disease as proposed by a recent publication that experimental inoculation with a strain of *S. chromogenes* VA654 by s.c. route induced EE in weaned pigs, which displayed an intraepidermal pustular dermatitis with exocytosis, exudation (crust formation), erosion, hyperkeratosis and acanthosis together with perivascular cellular infiltrations in dermis [17]. As *S. chromogenes* VA654 was originally isolated from the skin of a healthy pig, although the production of the exfoliative toxin (ExhB) could be detected at both DNA and protein levels, experimental inoculation of pigs with this strain only indicated the potential of *S. chromogenes* as a causative agent for EE. Since no field case of *S. chromogenes* infections in pigs has been described, the clinical evidence is still required to establish *S. chromogenes* as a pathogen for EE. Thus, no other species of Staphylococcus beyond *S. hyicus* has been previously described as a pathogen for EE in pigs. Here we report that a highly pathogenic strain of *Staphylococcus sciuri* HBXX06 caused an acute form of EE in piglets in both natural and experimental conditions, which suggested that the isolate of *S. sciuri* HBXX06 was a new emerging pathogen for EE in pigs.

## Results

### Fatal Exudative Epidermitis occurred in piglets

**Figure 1:**
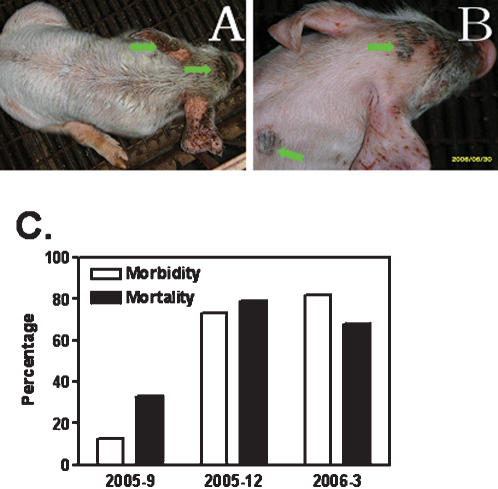
Clinical case of Exudative Epidermitis (EE) in piglets. A–B. Skin lesions (indicated by arrows) of five-day-old piglets with EE. C. Morbidity and mortality of suckling piglets from September of 2005 (n = 23), December of 2005 (n = 33) through March of 2006 (n = 95).

Exudative epidermitis occurred in the suckling piglets on a conventional pig farm with 172 sows in Hebei province in the fall of 2005 through May of 2006. The diseased piglets, which were born healthy, began to show clinical signs as early as 1 days following birth, displaying skin reddening, exfoliation, exudation and crusting, and succumbed to death within 2–4 days. The survivals were covered with brownish greasy layers (Figure 1A and 1B). As shown in Figure 1C, the mortality was 13% (3/23) in the beginning of the outbreak and reached up to 84% (80/95) within 6 months while the morbidity began with 33% (1/3) and reached up to 68% (54/80) in the spring of 2006. The nursing sow of diseased piglets manifested no other clinical signs than localized transient rashes or slight EE on the abdomen skin but recovered in a few days.

### A strain of *Staphylococcus sciuri carrying Exh-C* gene was isolated from a diseased piglet with EE

**Figure 2:**
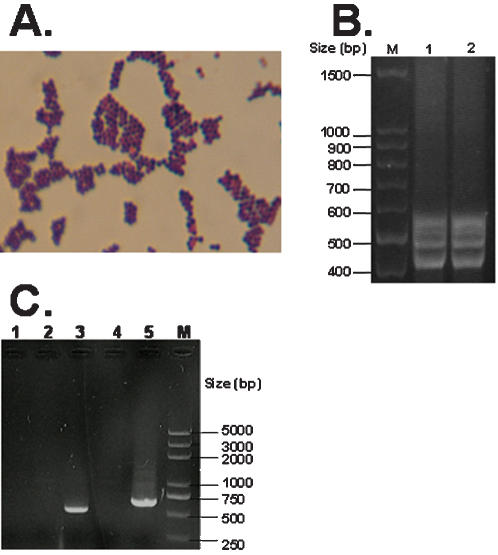
Gram's staining and molecular identification of Staphylococcus isolate. A. Gram's staining of the clinical Staphylococcus isolate. B. Identification of Staphylococcus subspecies by ITS-PCR analysis of ribosomal DNA in the isolates from natural EE (Lane 1) and experimental EE (Lane 2). C. Examination of exfoliative toxin gene of the Staphylococcus isolate by PCR. Lanes 1 through 5 represent the PCR products of Exh-A, Exh-B, Exh-C, Exh-D and 23s ribosomal DNA, respectively.

A strain of Staphylococcus, which we named HBXX06, was isolated in pure culture from the pericardial fluid of a moribund piglet and was identified as *S. sciuri* by morphological and biochemical examinations. The isolate displayed the morphology of *Staphylococcus* under the microscope, shaped as Gram staining positive cocci in clump (Figure 2A) and showed the biochemical characteristics of *S. sciuri*, which was up to 95% in common with the standard , but was totally different from that of *S. hyicus*. More importantly, this strain of staphylococcus was coagulase negative, resistant to novobiocin but sensitive to Polymycin B, which differentiated *S. sciuri* from *S. hyicus* (Table 2). These results indicated that the isolate HBXX06 was a strain of *S. sciuri*. As it has never been reported that *S. sciuri* could cause EE in pigs, this finding was quite unexpected. To confirm the results of biochemical examination, we further analyzed the strain *S. sciuri* HBXX06 at a molecular level using ITS-PCR assay and also examined the exfoliative toxins of the bacteria. As shown in Figure 2B, Strain HBXX06 showed four bands between 400 and 600 bp, which did not fit the ITS-PCR pattern of any coagulase-negative staphylococci tested by Couto and his colleagues [18]. This result suggested that *S. sciuri* HBXX06 may be a new subspecies of *S. sciuri*. Using specific primers for the amplification of exfoliative toxins, we found that HBXX06 carried Exh-C in its genome DNA (Figure 2C). In addition, specific band of 23s could also be amplified in the PCR assay, which confirmed the isolate as a member of *S. sciuri* at a molecular level (Figure 2C). Therefore, these results established the clinical isolate HBXX06 as an emerging member of *S. sciuri* group at both cellular and molecular levels.

**Table 1:**
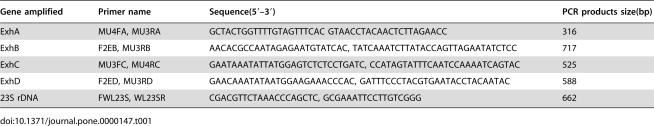
Oligonucleotide primers used in the PCR

Gene amplified	Primer name	Sequence(5′–3′)	PCR products size(bp)
ExhA	MU4FA, MU3RA	GCTACTGGTTTTGTAGTTTCAC GTAACCTACAACTCTTAGAACC	316
ExhB	F2EB, MU3RB	AACACGCCAATAGAGAATGTATCAC, TATCAAATCTTATACCAGTTAGAATATCTCC	717
ExhC	MU3FC, MU4RC	GAATAAATATTATGGAGTCTCTCCTGATC, CCATAGTATTTCAATCCAAAATCAGTAC	525
ExhD	F2ED, MU3RD	GAACAAATATAATGGAAGAAACCCAC, GATTTCCCTACGTGAATACCTACAATAC	588
23S rDNA	FWL23S, WL23SR	CGACGTTCTAAACCCAGCTC, GCGAAATTCCTTGTCGGG	662

**Table 2:**
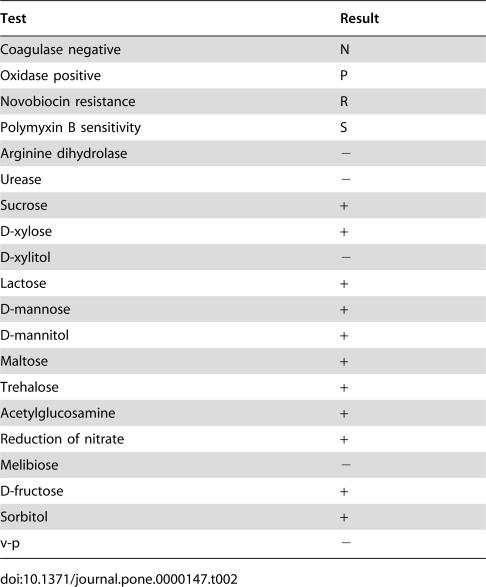
Biochemical traits of the clinical staphylococcus isolate, *S. sciuri* HBXX06

Test	Result
Coagulase negative	N
Oxidase positive	P
Novobiocin resistance	R
Polymyxin B sensitivity	S
Arginine dihydrolase	**−**
Urease	**−**
Sucrose	**+**
D-xylose	**+**
D-xylitol	**−**
Lactose	**+**
D-mannose	**+**
D-mannitol	**+**
Maltose	**+**
Trehalose	**+**
Acetylglucosamine	**+**
Reduction of nitrate	**+**
Melibiose	**−**
D-fructose	**+**
Sorbitol	**+**
v-p	**−**

### *S. sciuri* HBXX06 was highly pathogenic to piglets and mice

**Figure 3:**
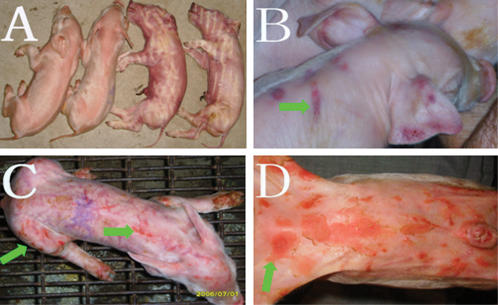
Reproduction of EE in newborn piglets inoculated with *S. sciuri* HBXX06 via *i.m.* injection or oral feeding. A. Four out of six newborn piglets treated with *S. sciuri* HBXX06 via *i.m.* injection or oral feeding at the dose of 1×10^10^ CFU per pig succumbed to death within 24 hours post infection. B–D. The skin lesions (indicated by arrows) of the survivals of the piglets on days 2, 3 and 4 respectively following oral feeding with 1×10^10^ CFU per piglet.

In order to determine whether the newly-isolated stain of *S. sciuri* HBXX06 was a causative agent for EE, we experimentally infected new born piglets with the isolate HBXX06 to reproduce the EE. As expected, the piglets inoculated with *S. sciuri* HBXX06 at a dose of 1×10^10^ CFU/head via oral feeding or *i.m.* injection showed fatal exudative epidermitis. All piglets in *i.m.* injection group (n = 3) died 12 hours post infection (Figure 3A). Piglets in oral feeding group started to show clinical signs of EE on the second day and died 4 days post infection (Figure 3B through 3D). The bacteria were recovered from the skin as well as the lung, lymph nodes and spleen of the infected piglets. The isolate showed the same biochemical traits (data not shown) and ITS-PCR pattern as the inoculums (Figure 2B). These results demonstrated that *S. sciuri* HBXX06 was highly pathogenic to the newborn piglets.

**Figure 4:**
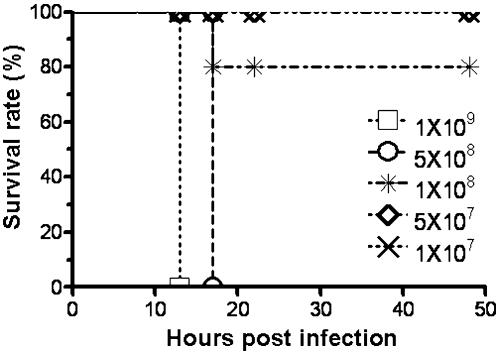
The survival rate of Balb/C mice post infection with *S. sciuri* HBXX06. Thirty 7-week old male Balb/C mice were divided into 6 groups with 5 mice each. Mice were infected with *S. sciuri* HBXX06 or PBS as controls as described in *Materials and Methods*. Results are representative of three experiments.

To determine whether the pathogenicity of *S. sciuri* HBXX06 to piglets could be generalized to other animals, we infected 7 weeks old Balb/C mice with the same bacterial isolate via *i.p.* injection. As shown in Figure 4, most of the mice receiving the dose of greater than 1×10^8^ CFU/mouse via *i.p.* injection died within 24 hour while the controls injected with PBS survived. These results clearly demonstrated that *S. sciuri* HBXX06 was highly pathogenic to piglets and mouse, which provided novel evidence that some strain of *S. sciuri* may cause EE in pigs in experimental conditions and the mice may be used as a model to investigate into the pathogenesis of *S. sciuri* HBXX06 in animals

## Discussion

Members of the *S. sciuri* group are widely distributed in nature, and they can be isolated from a variety of animals and the products of animal origin [19]–[21] as well as from human [22], [23], but most of them are apathogenic to animals. However they are important human pathogens responsible for?endocarditis [24], peritonitis [25], septic shock [26], urinary tract infection [27], pelvic inflammatory disease [28] and wound infections [29], [30]. Currently little information is known regarding the pathogenicity of *S. sciuri* in animals. It has been reported that some pathogenic strains of *S.sciuri* were responsible for mastitis in ruminants such as goats [31] and cow [32], which suggested that some members of *S.sciuri* possesses pathogenic potentials. In the present study, we reported that an isolate from an acute case of EE in piglets, identified as a member of *S. sciuri* group, was highly pathogenic to both piglets and mice.

Of note, the sows with diseased piglets displayed transient rashes and slight exudative epidermitis at abdomen before delivery, which suggested that the pathogenic strain of *S.sciuri* primarily transmit by contact. We hypothesized that the newborns might have picked up the pathogenic *S. sciuri* from contact with the mother when suckling or licking, by which large amount of the bacteria might have invaded the piglet by oral route. Therefore, in addition to inoculation of the animals by injection [17], we infected the newborn piglets via oral feeding. Our success in reproduction of fatal EE in piglet by oral infection strongly supported our assumption that the infection with *S.sciuri* HBXX06 by oral route might be the major mode of transmission for EE in piglets.

*S. hyicus* has been generally recognized as a causative agent for EE in pigs, however recently it has been reported that *S. chromogenes* could cause this disease in experimental condition[17]. Since no field case of *S. chromogenes* infections in pigs has been described, the clinical evidence is still required to establish *S. chromogenes* as a pathogen for EE in field condition. Our findings that *S. sciuri* HBXX06 could cause fatal EE in piglets in both field and experimental conditions greatly contributed to the further understandings of staphylococcus as pathogens responsible for EE in pigs.

The typical features of *S. sciuri* are coagulase negative, oxidase positive and novobiocin-resistant[33]–[35]. The isolate HBXX06 met these requirements. Besides, the PCR assay also confirmed the bacteria HBXX06 as a member of *S. sciuri* (Figure 2C). These results clearly indicated that the isolate HBXX06 is a member of *S. sciuri* family. However its ITS-PCR pattern did not match any of the coagulase negative staphylococci in the previous report [36], Which suggested that *S. sciuri* HBXX06 might have emerged as a new subspecies of *S. sciuri*. Since the *S. sciuri* group consists of *Staphylococcus sciuri* subsp. *carnaticus*, *Staphylococcus sciuri* subsp. *rodentium*, *Staphylococcus sciuri* subsp. *sciuri*, *Staphylococcus lentus*, and *Staphylococcus vitulinus* [37], [38], more efforts will be required to elucidate the genotype of this isolate.

Exfoliative toxins are critical factors in the pathogenesis of EE in pigs. The genes encoding four different exfoliative toxin from *S. hyicus* (ExhA, ExhB, ExhC, and ExhD) were cloned and sequenced [39]. The presence of different exfoliative toxins in staphylococci could be examined using PCR. It was shown that ExhA, ExhB, ExhC and ExhD had been detected in 20, 33, 18 and 22%, respectively, of 60 cases of EE investigated [40]. It was also reported that one or more exfoliative toxins produced by isolates of *S. hyicus* from different countries could be detected [6]. Dakic and his colleagues examined the genes encoding staphylococcal enterotoxins (sea to see, seg, and seh), toxic shock syndrome toxin 1 (tst), and exfoliative toxins (eta and etb) in a panel of 48 *Staphylococcus sciuri* group isolates from animals and did not find any these genes [41]. In Another study, one hundred and twenty-one isolates from human and animals were examined for biofilm formation, hemagglutination, presence of clumping factor, production of spreading factors and exotoxins, cytotoxicity and capacity to stimulate nitric oxide production, and it was found that a wide spectrum of possible virulence determinants of *S. sciuri* existed [42], however their exact contribution to virulence of this bacterium in vivo remains to be determined. In the present study, we showed that *S. sciuri* HBXX06 harbored the gene encoding Exh-C (Figure 2C). These results suggest that the Exh-C might be one of these toxic genes involved in pathogenesis of EE piglets infected with *S. sciuri* HBXX06. As such, several questions need to be addressed, for example, where did the pathogenic strain of *S. sciuri* HBXX06 come from? What virulent factors *S. sciuri* HBXX06 has? What are their exact roles in the pathogenesis of acute EE in piglets? and what is the relationship of this bacterium to *S.hyicus*, the causative agent for EE in pigs?

In summery, we isolated an emerging strain of *S. sciuri* from the pericardial fluid of a diseased piglet with EE and succeeded in reproduction of fatal EE in piglet with the isolate, which clearly established that some member of *S. sciuri* may act as an important causative agent to cause fatal EE in piglets. These findings prioritized *S. sciuri* as important zoonotic agents, which may have ramifications for human medicine.

## Materials and Methods

### Investigation of the EE's epidemiology on the farm

Clinical symptoms of EE were recorded based on the gross lesions and necropsy of the piglets from a conventional pig-rearing farm in Hebei province of China. Diseased pigs were sacrificed for pathological examination and isolation of pathogens following euthanasia. All procedures were approved by the animal care and use committee of China Agricultural University.

### Aetiological isolation and inoculum preparation

Swab samples were collected from the pericardial fluid of a diseased piglet with EE. The isolate was grown aerobically on nutrient agar (Land Bridge Technology Co., Ltd, Beijing) or Manitol salt agar base medium (Aoboxing Universeen Bio-Tech CO., LTD, Beijing) at 37°C for 17–24 h. The isolate was replicated in Nutrient Broth Medium (Aoboxing Universeen Bio-Tech CO., LTD, Beijing). Log-phase growing cultures were washed twice with PBS and stored at −70°C until use.

### Identification of the clinical isolate

(i) Cellular identification of the Staphylococcus isolate by biochemical characteristics and antibiotic sensitivity test: The clinical isolate was examined for members of the family Micrococcaceae by means of the colony morphology and pigmentation, Gram's staining and catalase test, and further tested for biochemical traits including oxidase (Land Bridge Technology Co., Ltd, Beijing) and coagulase production, Novobiocin and Polymyxin B sensitivity and others using TH-16S bacteria coding system (Beijing Land Bridge Technology, Beijing). (ii) Molecular identification of the Staphylococcus isolate by ITS-PCR: DNA was isolated via commercial DNA Extraction Kit per manufacturer's instruction (TianGen Biotech (Beijing) Co., Ltd, Beijing). The ITS-PCR was performed as previously described [43] with some modifications, using primers G1 (5-GAAGTCGTAACAAGG) and L1 (5-CAAGGCATCCACCGT) (Shanghai Sangon Biological Engineering & Technology and Service Co., Ltd, Beijing). Amplification reaction was performed on Techne TC512 Gene Amp PCR System (Techne Corporation, UK). The program consisted of an initial denaturation step at 94°C for 5 min and 30 amplification cycles, each with 30 seconds at 94°C, 1 min at 55°C and 1 min at 72°C, followed by an additional extension step at 72°C for 10 min. Amplification products were resolved for 2 h in 2% agarose gels (ShanghaiAiyito Bio-instrument Co., Ltd) in TAE buffer containing 0.04 M Tris-acetate and 0.001 M EDTA (pH 8), after supplemented with 0.06µl of SYBR Green I Loading buffer (Beijing SBS Genetech Co., Ltd, Beijing) per ml. The standard DNA ladder marker was purchased from TianGen Biotech Company (Beijing). After photography by AlphaImager (Alpha Innotech, Beijing), DNA patterns were visually analyzed [44].

### Examination of genes encoding Staphylococcus of exfoliative toxins by PCR

The PCR was performed as previously described [45], using primers listed in Table 1. DNA was amplified by an initial denaturation at 94°C for 5 min followed by 30 cycles of 30 seconds at 94°C, 1 min at 56°C and 30 seconds at 72°C. The PCR reaction was completed by 10 min incubation at 72°C for the full extension of the PCR products. Amplification products were resolved in 2% agarose gel with the standard DNA ladder markers (TianGen Biotech (Beijing) Co., Ltd, Beijing).

### Experimental infection in mice

Thirty 7-week old male Balb/C mice were divided into 6 groups with 5 mice each. Six groups of mice were inoculated respectively with the bacterial isolate at the dose of 1×10^9^ CFU, 5×10^8^ CFU, 1×10^8^ CFU, 5×10^7^ CFU, 1×10^7^ CFU and 1×10^6^ CFU in 0.2 ml of sterile Phosphorus saline (PBS) per mouse via intraperitoneal injection. Livers, spleens, kidney and pancreases were removed from the infected mice at different time points after *bacterial* infection, and organ homogenates were used for the examination of bacterial presence [46].

### Experimental infection in pigs

Three out of nine newborn Changbai piglets in the same litter were experimentally infected with the clinical isolate via intra-muscular injection, another three were infected by oral feeding route and the rest three were injected with sterile PBS as the controls. The piglets were not allowed to suckle following birth until 45 minutes post infection. Cotton swab samples from the skin behind the right ear were collected from each of the experimental animals for microbiological examination. Each piglet in infection groups received 1×10^10^ CFU of the bacterial isolates in 1 ml sterile PBS either via *i.m.* injection or oral feeding route. The piglets were clinically observed throughout the experiment. Clinical signs were recorded and necropsy was performed immediately after euthanasia. Samples for aetiological examination were collected from affected skin, liver, spleen and kidney from each pig and plated on Manitol Salt Agar base medium. Colony on the Manitol Salt Agar was then subjected to identification by biochemical test and also by PCR for the presence of exfoliative toxins and ITS PCR analysis of 23S ribosomal DNA as described above.
